# Caoutchouc and Gutta-Percha Cements

**Published:** 1898-06

**Authors:** 


ARTICLE VIII.

### CAOUTCHOUC AND GUTTA-PERCHA CEMENTS.

A gutta-percha cement for leather is obtained by melting together 100 parts of gutta-percha, 200 parts asphalt or pitch, and 15 parts oil of turpentine, It is to be used hot.

Elastic gutta-percha cement, especially for fixing soles to shoes, which does not crack in bending, on account of its great extensibility, is prepared by dissolving 10 parts gutta-percha in 100 parts benzine and pouring the solution into 100 parts linseed oil varnish, shaking well. The leather must be roughened before using this cement, in order to insure greater durability. By a caseine-borax cement a handsome surface gloss is imparted to the leather. The borax is dissolved in boiling water and the borax solution poured into freshly prepared caseine. The durable thick cement is very servicable.

Good caoutchouc cements, for rubber strips or rubber goods on metal, are obtained by dissolving shellac in ten times its weight of ammonia. After standing for three to four weeks a transparent putty results, which is used without heating. The cemented places soften at first, but becomes hard and firm after evaporation of the ammonia, which may be assisted by heating. This cement is water-tight and gas-proof, and is also useful for hard rubber articles. A cement made of a mixture of gutta-percha with asphalt is serviceable for the same purpose. This has to be applied hot and the pieces are to be pressed together.

Very useful cement for leather belting is manufactured by kneading 10 parts carbon bisulphid and 1 part of oil of turpentine with gutta-percha till a thick paste results. The portions of the leather where the cement is to be applied must be unoiled and roughened; the cement is put on and the ends are pressed together till the binding agent has become dry. Directions for caoutchouc cement are : 100 parts finely cut caoutchouc, 15 parts rosin, 20 parts shellac, dissolved in sulphid of carbon. One part caoutchouc, 7 parts mastic, and 50 parts chloroform, left to stand several weeks.

Cement for rubber boots, etc : (1) 10 parts caoutchouc dissolved in 250 parts chloroform; (2) 10 parts caoutchouc, 4 parts rosin, 40 parts oil of turpentine, mixed and dissolved. For use, pour together equal parts of both solutions. Both ladies' and gentlemen's shoes may be soled by sheets prepared especially and gummed on with this cement.—Translated from the *Farben Zeitung*.

